# Would Chinese Men Who Have Sex With Men Take Up Human Papillomavirus (HPV) Screening as an Alternative Prevention Strategy to HPV Vaccination?

**DOI:** 10.3389/fmed.2022.904873

**Published:** 2022-06-03

**Authors:** Paul Shing-fong Chan, Yuan Fang, Andrew Chidgey, Francois Fong, Mary Ip, Zixin Wang

**Affiliations:** ^1^Jockey Club School of Public Health and Primary Care, Faculty of Medicine, The Chinese University of Hong Kong, Hong Kong, Hong Kong SAR, China; ^2^Department of Health and Physical Education, The Education University of Hong Kong, Hong Kong, Hong Kong SAR, China; ^3^AIDS Concern, Hong Kong, Hong Kong SAR, China; ^4^Neohealth, Hong Kong, Hong Kong SAR, China

**Keywords:** HPV screening, HPV vaccination, men who have sex with men, CBO-private clinic service model, online health promotion

## Abstract

**Background:**

Men who have sex with men (MSM) are at high risk for human papillomavirus (HPV) infection. A community-based organization (CBO)-private clinic service model promoting HPV vaccination among MSM was implemented in Hong Kong. The aim of this study was to evaluate the effectiveness of this service model in increasing HPV screening among MSM.

**Methods:**

This was a secondary analysis of the CBO-private clinic service model in increasing HPV screening among MSM. Participants were Hong Kong Chinese-speaking MSM aged 18–45 years who had never received HPV vaccination. All participants completed a telephone survey at baseline before receiving online intervention promoting HPV vaccination and completed another telephone survey 12 months afterward.

**Results:**

A total of 350 participants completed a baseline telephone survey and received interventions promoting HPV vaccination. Among 274 participants being followed up at Month 12, 33 (12.0%) received any type of HPV screening during the study period. Such uptake rate was similar to the prevalence of HPV screening in the past year measured at baseline (12.0 vs. 9.9%, *p* = 0.43). More MSM preferred HPV vaccination or HPV vaccination plus HPV screening, and very few preferred HPV screening alone. After adjusting for significant baseline characteristics, higher perceived susceptibility to HPV (adjusted odds ratio (AOR): 1.16, 95% confidence interval (CI): 1.00–1.34) and receiving HPV vaccination during the study period (AOR: 7.03, 95% CI: 3.07–16.13) were significantly associated with higher HPV screening uptake.

**Conclusions:**

The CBO-private clinic service model promoting HPV vaccination had limited impact in increasing HPV screening among MSM in Hong Kong. MSM in Hong Kong may not use HPV screening as an alternative prevention strategy to HPV vaccination. Future programs preventing HPV-related diseases among MSM in Hong Kong should focus on HPV vaccination promotion.

## Introduction

Men who have sex with men (MSM) are at high risk for human papillomavirus (HPV) infection (1). Globally, 92.6 and 63.9% of MSM with and without human immunodeficiency virus (HIV) infection had genital HPV infection, respectively (2). The prevalence of HPV infection was 66.3% among Chinese MSM (3). MSM had a much higher prevalence of HPV infection than the male general population (4–6). Along with the high prevalence of genital warts (7), MSM had a 32–52 times higher risk of developing anal cancer than the general population (8). Over the past 20–30 years, the incidence of squamous cell anal cancers increased by about 96% in men in the United States, 91% of which were caused by persistent infection of HPV types 16 and/or 18 (9–11). HIV-infected MSM had the highest HPV-related cancer risk, which accounted for 9.9% of Chinese MSM in 2016 (12).

HPV screening and HPV vaccination are considered as two important strategies for preventing anal cancers among MSM (10). On one hand, studies revealed that regular screening for HPV and anal cancer increased the quality-adjusted life expectancy of MSM and was considered as a cost-effective strategy (13–15). HPV screening every 2–3 years for MSM is recommended by some international health authorities (16, 17). In recent years, HPV screening has been adopted as a standard intervention for MSM by some practitioners (18). On the other hand, HPV vaccination is highly effective in preventing vaccine-type genital warts, cancers, low-grade anal intraepithelial neoplasia (AIN1) and cervical intraepithelial neoplasia (CIN1) (19, 20). The United States Centers for Disease Control and Prevention (CDC) recommends HPV vaccination for MSM aged ≤ 45 years (21, 22). Free or subsidized HPV vaccination programs have been implemented in some countries for MSM, such as the United Kingdom (23, 24), Australia (25), and the United States (26). In Hong Kong where this study was conducted, there was no governmental HPV/anal cancer screening or HPV vaccination program targeting MSM. Public hospitals or social hygiene clinics provide free HPV screening for men (27). Free or chargeable HPV screening services are available to MSM in some community-based organizations (CBOs) (28). MSM can also seek chargeable HPV screening (about HK$700 or US$90 per episode) and HPV vaccination (about HK$4,600 or US$593 for 3 doses) services at private hospitals/clinics. However, the utilization of HPV screening (19.1% in lifetime, and 9.7% in the past year) or HPV vaccination (almost 0%) was very low among MSM in Hong Kong (29–31).

A CBO-private clinic service model promoting HPV vaccination among MSM was implemented in Hong Kong (32). Experienced staff from a CBO with good access to MSM approached MSM aged 18–45 years who had never received HPV vaccination, invited them to join the study, and provided them with online health promotion, and interested MSM were referred to gay-friendly private clinics for HPV vaccination. Although a 10% discount from market price (about HK$4,200 or US$542 for 3 doses) was offered to MSM in the service model, only 16.8% of them had taken up HPV vaccination as many MSM still considered the cost to be unaffordable. The online intervention promoting HPV vaccination contained health communication messages about the risk and severity of HPV infection (32), which were associated with higher intention to receive HPV screening among local MSM (29). Such online health promotion might motivate local MSM to receive HPV screening. It is possible that some MSM who did not take up HPV vaccination would prefer using HPV screening as a cheaper HPV prevention strategy.

This study was a secondary analysis of the aforementioned CBO-private clinic service model promoting HPV vaccination among MSM in Hong Kong (32). The objective was to evaluate the effectiveness of such service model in increasing utilization of HPV screening within a 12-month follow-up period. We compared the HPV screening uptake during the follow-up period with the uptake rate within the past year measured at baseline survey to determine the effectiveness. Baseline factors predicting HPV screening uptake were investigated. In addition, this study investigated whether MSM would use HPV screening as an alternative to HPV vaccination.

## Methods

### Study Design

**Figure 1:**
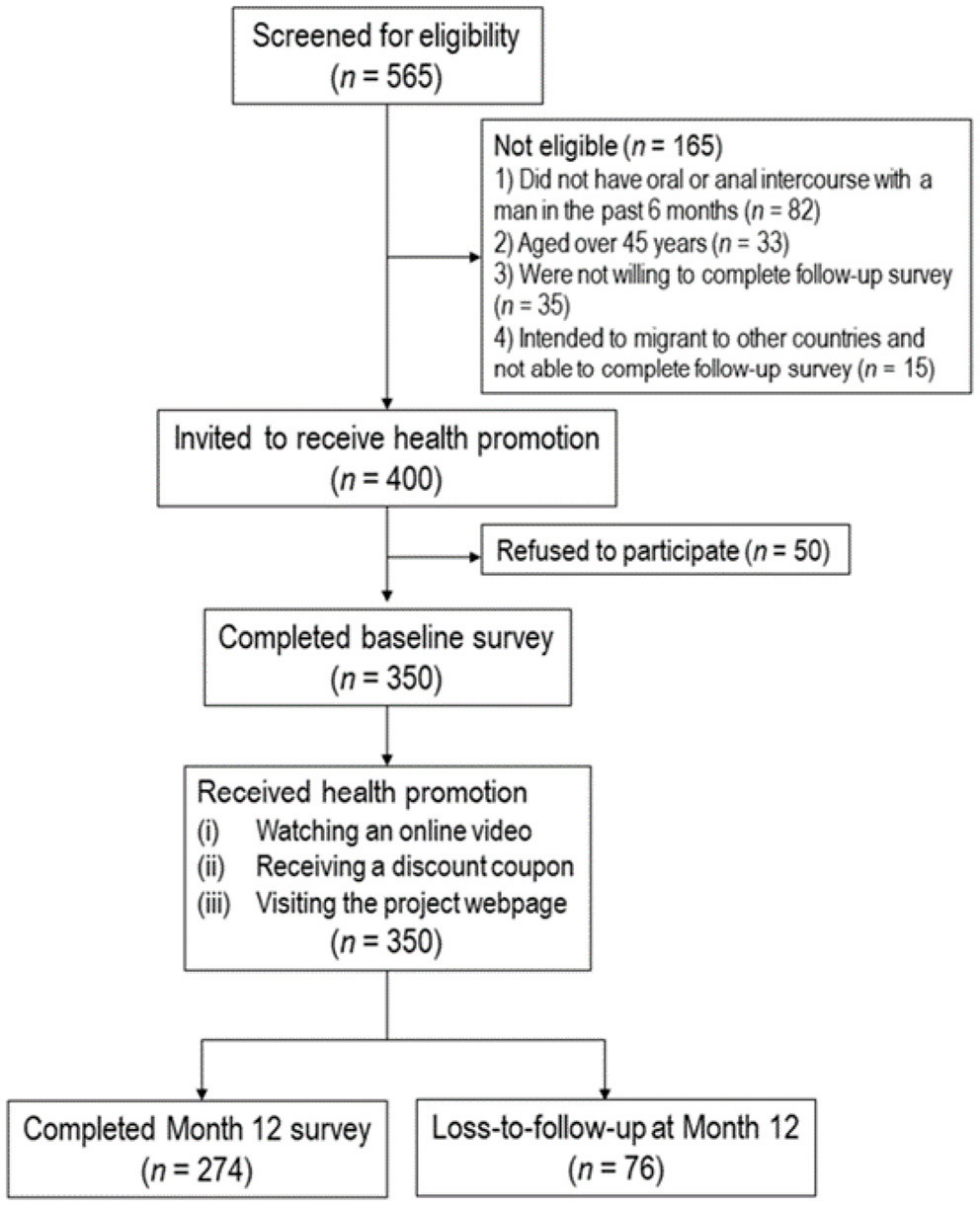
Flowchart diagram of the study.

This was a secondary analysis of a longitudinal study promoting HPV vaccination among MSM in Hong Kong, China which was conducted from August 2019 to April 2021 (32). All participants completed a telephone survey at baseline before receiving online intervention promoting HPV vaccination and completed another telephone survey 12 months after the baseline survey. There was no control or comparison group. The flow chart diagram was shown in Figure 1. The original study was registered at ClinicalTrial.gov (NCT04815837).

### Participants

Inclusion criteria of the original longitudinal study were: (1) Hong Kong Chinese-speaking males aged 18–45 years, (2) self-reported having had oral or anal intercourse with at least one man in the last 6 months, (3) never received HPV vaccination, and (4) willing to complete a follow-up telephone survey 12 months after the baseline survey.

### Sampling and Data Collection

Recruitment details were reported in previously published articles (29, 32). Trained and experienced staff of a collaborative CBO providing HIV-related services worked as fieldworkers for recruitment. Participants were recruited through multiple sources, including outreaching in gay bars or saunas in Hong Kong, online recruitment, and referrals made by peers and other CBOs. Participants were guaranteed anonymity during the study, and they had the right to withdraw from the study at any time. Their refusal or withdrawal from the study would not affect their access to any future services. Verbal instead of written informed consent was obtained due to maintain anonymity, and the fieldworkers signed a form pledging that the participants had been fully informed about the study. Multiple forms of contact information were obtained to make appointments for conducting the baseline and Month 12 telephone survey. A HK$25 (approximately US$3.2) supermarket or café coupon was mailed to participants as compensation for their time after they completed each survey. Among 400 eligible participants being approached, 50 refused to participate in the study for time and/or other logistic reasons, and 350 (87.5%) completed the baseline survey. A total of 274 participants (78.3%) completed the follow-up survey.

### The CBO-Private Clinic Service Model Promoting HPV Vaccination

A CBO providing HIV prevention services to local MSM and a group of gay-friendly private clinics developed the service model. In the service model, CBO staff approached MSM through outreaching in gay bars or saunas, online recruitment, and peer referral. After completion of a baseline telephone survey, participants received health promotion with the following components: (1) watching an online video promoting HPV vaccination for MSM, which talked about the high risk of penile/anal cancers among MSM, severe consequences of HPV-related diseases, benefit of HPV vaccination, and the convenient and gay-friendly procedures to receive HPV vaccination in the collaborative private clinics, (2) receiving a 10% discount coupon to receive HPV vaccination, (3) accessing a project webpage, and (4) receiving five reminders for HPV vaccination uptake. All participants of the service model completed a follow-up telephone survey at Month 12.

### Measures

#### Primary Outcome: HPV Screening Uptake

Uptake of HPV screening in the past year was measured at both baseline and Month 12. Participants who received HPV screening during the follow-up period were asked about some details (location, cost, and results of HPV screening). The primary outcome was validated by requesting participants to send an image of their receipt of HPV screening hiding personal identification to the project staff.

#### Predictor: HPV Vaccination Uptake During the Follow-Up Period

Participants reported the number of doses of HPV vaccination received during the follow-up period. To validate HPV vaccination uptake, participants were requested to send an image of their receipt of HPV vaccination hiding personal identification to the project staff. Uptake of any dose of HPV vaccination during the follow-up period was used as a predictor.

#### Predictors: Baseline Background Characteristics

Information collected included socio-demographics, sexual orientation, HIV or sexually transmitted infection (STI) prevention service utilization, history of HIV, and other STIs. Queried sexual behaviors included anal intercourse with male regular/ non-regular sex partners and male sex workers, condomless anal intercourse (CAI) with men, multiple male sex partnerships, sexualized drug use (SDU), and so on. Regular male sex partners (RP) were defined as lovers and/or stable boyfriends, while non-regular male sex partners (NRP) were defined as casual sex partners. In this study, SDU was defined as the use of any psychoactive substance before or during sexual intercourse (33, 34).

#### Predictors: Perceptions Related to HPV Screening in General Based on the Health Belief Model (HBM) at Baseline

Four scales derived from the HBM were constructed. They were: (1) the three-item Perceived Susceptibility Scale, (2) the four-item Perceived Severity Scale, (3) the two-item Perceived Benefit Scale, and (4) the two-item Perceived Barrier Scale. The response categories for these scales were: 1 = strongly disagree, 2 = disagree, 3 = neutral, 4 = agree, and 5 = strongly agree. The Cronbach's alpha of these scales ranged from 0.71 to 0.85. Single factors were identified by the exploratory factor analysis, explaining 54.9–77.9% of the total variance. Additionally, two single items measuring Perceived Cue to Action and Perceived Self-Efficacy were constructed.

#### Predictors: Behavioral Intention to Take Up Any Type of HPV Screening at Baseline

Two single items measured perceived chance to take up clinician-collected HPV screening in the next year if it costs HK$700 (US$89.7) per episode and perceived chance to take up clinician-collected HPV screening in the next year if it is provided for free. The response categories for these items: were 1 = very low, 2 = low, 3 = moderate, 4 = high, and 5 = very high. They were treated as two independent variables.

### Ethics Statement

Ethics approval was obtained from the Survey and Behavioral Research Ethics Committee of the Chinese University of Hong Kong (reference number: KPF18HLF22).

### Statistical Analysis

The difference in baseline characteristics between those who were followed up at Month 12 and those who were lost to follow-up were compared using the chi-square test (X^2^) for categorical variables and independent-sample *t*-test for continuous variables. Among participants who completed both surveys, we compared validated HPV screening uptake during the follow-up period with that measured at baseline survey using McNemar test. Using validated uptake of HPV screening during the follow-up period as the dependent variable, and sociodemographic characteristics as independent variables, crude odds ratio (OR) predicting the dependent variable were obtained using logistic regression models. After adjusting for those sociodemographic characteristics with *p* < 0.05 in the univariate analysis, the associations between the independent variables of interest (e.g., perceptions related to HPV screening) and the dependent variable were then obtained by adjusted odds ratio (AOR), and respective 95% confidence interval (CI) were derived from the analyses. Each AOR was obtained by fitting a single logistic regression model, which involved one of the independent variables and the significant sociodemographic variables. SPSS version 26.0 (Chicago, IL, USA) was used for data analysis, and *p*-values < 0.05 were considered as statistically significant.

## Results

### Baseline Characteristics

At baseline, over half of the participants were aged 25–40 years (74.6%), currently single (75.7%), had attained tertiary education or above (86.6%), full-time employed (82.9%), had monthly income of less than HK$40000 (US$5,129) (79.2%), identified themselves as homosexual (91.1%), had never smoked (73.4%), drank in the past year (75.4%), did not take up HPV screening in the past year (90.3%). Regarding history of HIV, other STIs, and service utilization, 1.7% self-reported being HIV positive, 23.1% had ever been infected by other STIs, and 23.1% had utilized other prevention services (e.g., receiving free condoms, peer education, and pamphlets, and attending seminars). The prevalence of anal intercourse with regular male sex partners, non-regular male sex partners, and male sex workers were 83.7, 48.6, and 2.6%, respectively, whilst 50.3% had CAI with men. The prevalence of multiple male sex partnerships, sexual intercourse with female sex partners, and condomless sex with female sex partners was 54.6, 1.7, and 0.9%, respectively, and 5.7%% had sexualized drug use (Table 1).

**Table 1:** Baseline characteristics of the participants.

	**All participants (*n* = 350)**	**Being followed up at month 12 (*n* = 274)**	**Dropouts (*n* = 76)**	***P*-values**
	***n* (%)**	***n* (%)**	***n* (%)**	
**Socio-demographics**				
**Age group (years)**				
18–24	48 (13.7)	34 (12.4)	14 (18.4)	0.34
25–30	128 (36.6)	106 (38.7)	22 (28.9)	
31–40	133 (38.0)	103 (37.6)	30 (39.5)	
>40	41 (11.7)	31 (11.3)	10 (13.2)	
**Relationship status**				
Currently single	265 (75.7)	210 (76.6)	55 (72.4)	0.44
Married or cohabited with a man	85 (24.3)	64 (23.4)	21 (27.6)	
**Highest education level attained**				
Secondary or below	47 (13.4)	37 (13.5)	10 (13.2)	0.94
Tertiary of above	303 (86.6)	237 (86.5)	66 (86.8)	
**Employment status**				
Full-time	290 (82.9)	229 (83.6)	61 (80.3)	0.50
Part-time/unemployed/retired/students	60 (17.1)	45 (16.4)	15 (19.7)	
**Monthly personal income, HK$ (US$)**				
<10,000 (1,282)	37 (10.6)	27 (9.9)	10 (13.2)	0.28
10,000–19,999 (1,282–2,564)	106 (30.3)	87 (31.8)	19 (25.0)	
20,000–39,999 (2,565–5,128)	134 (38.3)	107 (39.1)	27 (35.5)	
40,000 or above (5,129)	67 (19.1)	50 (18.2)	17 (22.4)	
Refuse to disclose	6 (1.7)	3 (1.1)	3 (3.9)	
**Sexual orientation**				
Homosexual	319 (91.1)	252 (92.0)	67 (88.2)	0.30
Bisexual	31 (8.9)	22 (8.0)	9 (11.8)	
**Lifestyles**				
**Smoking in lifetime**				
No	257 (73.4)	203 (74.1)	54 (71.1)	0.60
Yes	93 (26.6)	71 (25.9)	22 (28.9)	
**Drinking in the past year**				
No	86 (24.6)	64 (23.4)	22 (28.9)	0.32
Yes	264 (75.4)	210 (76.6)	54 (71.1)	
**History of HPV screening**				
**HPV screening in the past year**				
No	316 (90.3)	247 (90.1)	69 (90.8)	0.87
Yes	34 (9.7)	27 (9.9)	7 (9.2)	
**History of HIV and other sexually transmitted infections (STI) and service utilization**				
**Self-reported HIV sero-status**				
Negative	311 (88.9)	251 (91.6)	60 (78.9)	0.02
Positive	6 (1.7)	4 (1.5)	2 (2.6)	
Refuse to disclose	11 (3.1)	7 (2.6)	4 (5.3)	
Had never tested for HIV antibody	22 (6.3)	12 (4.4)	10 (13.2)	
History of other STIs (Yes)	81 (23.1)	67 (24.5)	14 (18.4)	0.27
Utilization of other HIV/STI prevention services (e.g., receiving free condoms, peer education and pamphlets, and attending seminars) (Yes)	81 (23.1)	69 (25.2)	12 (15.8)	0.09
**Sexual behaviors in the past 6 months**				
Anal intercourse with regular male sex partners (Yes)	293 (83.7)	230 (83.9)	63 (82.9)	0.83
Anal intercourse with non-regular male sex partners (Yes)	170 (48.6)	124 (45.3)	46 (60.5)	0.02
Anal intercourse with male sex workers (Yes)	9 (2.6)	4 (1.5)	5 (6.6)	0.01
Condomless anal intercourse with men (Yes)	176 (50.3)	142 (51.8)	34 (44.7)	0.27
Multiple male sex partnerships (Yes)	191 (54.6)	143 (52.2)	48 (63.2)	0.09
Sexual intercourse with female sex partners (Yes)	6 (1.7)	3 (1.1)	3 (3.9)	0.09
Condomless sex with female sex partners (Yes)	3 (0.9)	2 (0.7)	1 (1.3)	0.62
Sexualized drug use (use of psychoactive substances before or during sexual intercourse) (Yes)	20 (5.7)	17 (6.2)	3 (3.9)	0.45
**Perceptions related to HPV screening (in general) based on the HBM**				
**Perceived susceptibility to HPV (high/very high)**				
Perceived risk of contracting HPV in lifetime	89 (25.4)	65 (23.7)	24 (31.6)	0.16
Perceived risk of contracting genital warts in lifetime	72 (20.6)	50 (18.2)	22 (28.9)	0.04
Perceived risk of having penile/anal cancers in lifetime	42 (12.0)	32 (11.7)	10 (13.2)	0.73
Perceived Susceptibility Scale ^a^, mean (SD)	8.2 (2.6)	8.1 (2.6)	8.8 (2.7)	0.35
				
**Perceived severity of HPV-related diseases (agree/strongly agree)**				
HPV infection would increase risk of HIV acquisition	106 (30.3)	81 (29.6)	25 (32.9)	0.58
HPV infection would cause penile or anal cancers	127 (36.3)	98 (35.8)	29 (38.2)	0.70
Genital warts would have severe harms on your health	182 (52.0)	140 (51.1)	42 (55.3)	0.52
Penile or anal cancers would have severe harms on your health	263 (75.1)	207 (75.5)	56 (73.7)	0.74
Perceived Severity Scale ^b^, mean (SD)	13.4 (3.1)	13.3 (3.2)	13.8 (2.7)	0.43
**Perceived benefits of HPV screening (agree/strongly agree)**				
HPV screening could detect HPV infection earlier, so as to have better treatment outcomes	314 (89.7)	243 (88.7)	71 (93.4)	0.23
HPV screening could prevent cancers caused by HPV infection	273 (78.0)	211 (77.0)	62 (81.6)	0.40
Perceived Benefit Scale ^c^, mean (SD)	8.5 (1.5)	8.5 (1.6)	8.5 (1.2)	0.97
Perceived barriers of receiving HPV screening (agree/strongly agree)				
Others would think you are having high risk sexual behaviors	57 (16.3)	40 (14.6)	17 (22.4)	0.11
HPV screening would cause pain and discomfort	38 (10.9)	24 (8.8)	14 (18.4)	0.02
Perceived Barrier Scale ^d^, mean (SD)	4.9 (1.7)	4.8 (1.5)	5.1 (2.2)	0.19
Perceived cue to action related to HPV screening (agree/strongly agree)				
People who are important to you suggest that you take up HPV screening	148 (42.3)	117 (42.7)	31 (40.8)	0.77
Item score, mean (SD)	3.3 (1.1)	3.3 (1.1)	3.2 (1.1)	0.50
**Perceived self-efficacy related to HPV screening (agree/strongly agree)**				
It is easy for you to take up HPV screening in the next year if you want to	189 (54.0)	146 (53.3)	43 (58.6)	0.61
Item score, mean (SD)	3.6 (1.0)	3.6 (1.0)	3.7 (0.9)	0.47
**Behavioral intention to take up any type of HPV screening**				
**Perceived chance to take up clinician-collected HPV screening in the next year if it cost HK $700 (US $89.7) per episode**				
Very low/low/moderate	280 (80.0)	217 (79.2)	63 (82.9)	0.48
High/very high	70 (20.0)	57 (20.8)	13 (17.1)	
**Perceived chance to take up clinician-collected HPV screening in the next year if it is provided for free**				
Very low/low/moderate	81 (23.1)	60 (21.9)	21 (27.6)	0.29
High/very high	269 (76.9)	214 (78.1)	55 (72.4)	

a * Perceived Susceptibility Scale: three items, Cronbach's alpha: 0.85, one factor was identified by exploratory factor analysis, explaining for 77.2% of total variances*.

b *Perceived Severity Scale: four items, Cronbach's alpha: 0.72, one factor was identified by exploratory factor analysis, explaining for 54.9% of total variances*.

c *Perceived Benefit Scale: two items, Cronbach's alpha: 0.71, one factor was identified by exploratory factor analysis, explaining for 77.9% of total variances*.

d *Perceived Barrier Scale: two items, Cronbach's alpha: 0.75, one factor was identified by exploratory factor analysis, explaining for 57.4% of total variances*.

*HBM, Health Belief Model; HIV, Human Immunodeficiency Virus; HPV, Human Papillomavirus; SD, Standard Deviation; STI, Sexually Transmitted Infection*.

When comparing those who were followed up (*n* = 274) and were lost to follow-up (*n* = 76), significant differences were found in several background characteristics which included self-reported HIV sero-status (*p* = 0.02), anal intercourse with NRP (*p* = 0.02), anal intercourse with male sex workers (*p* = 0.01), perceived risk of contracting genital warts in lifetime (*p* = 0.04), and perceived HPV screening would cause pain and discomfort (*p* = 0.02) (Table 1).

### Uptake of HPV Screening

Among the 274 participants who were followed up at Month 12, 12.0% (*n* = 33) received any type of HPV screening. Such uptake rate was similar to the prevalence of HPV screening in the past year measured at baseline (12.0 vs. 9.9%, *p* = 0.43). Among the 33 participants who received any HPV screening during the project period, 3.0% (*n* = 1) were diagnosed with HPV infection in genital, 9.1% (*n* = 3) in both genital and anus, and 6.1% (*n* = 2) in other locations. Regarding their most recent episode of HPV screening, 15.2% (*n* = 5) were taken place in public hospitals, 63.6% (*n* = 21) in private clinics, 15.2% (*n* = 5) in CBOs, 3.0% (*n* = 1) in other organizations, and 3% (*n* = 1) by self-collected screening. For the amount spent on the most recent episode of HPV screening, 69.7% (n=23) received free HPV screening, while 30.3% (*n* = 10) spent HK$30–2000 (US$3.8–256.6).

### Factors Associated With the Uptake of HPV Screening During the Follow-Up Period

In univariate analysis, participants who took HPV screening in the past year, and self-reported as HIV positive at the baseline were more likely to take up HPV screening at the follow-up period (Table 2).

**Table 2:** Baseline background characteristics predicting uptake of any HPV screening (among participants who completed Month 12 follow-up, *n* = 274).

	**OR (95%CI)**	***P*-values**
**Socio-demographics**		
**Age group (years)**		
18–24	1.0	
25–30	0.54 (0.20, 1.49)	0.23
31–40	0.37 (0.13, 1.08)	0.07
>40	0.57 (0.15, 2.18)	0.41
**Relationship status**		
Currently single	1.0	
Married or cohabited with a man	0.30 (0.09, 1.00)	0.05
**Highest education level attained**		
Secondary or below	1.0	
Tertiary of above	1.15 (0.38, 3.48)	0.80
**Employment status**		
Full-time	1.0	
Part-time/unemployed/retired/students	0.90 (0.33, 2.46)	0.83
**Monthly personal income, HK$ (US$)**		
<10,000 (1,282)	1.0	
10,000–19,999 (1,282–2,564)	1.44 (0.29, 7.12)	0.65
20,000–39,999 (2,565–5,128)	2.20 (0.47, 10.20)	0.32
40,000 (5,129)	1.71 (0.32, 9.09)	0.53
Refuse to disclose	N.A.	N.A.
**Sexual orientation**		
Homosexual	1.0	
Bisexual	2.35 (0.81, 6.87)	0.12
**Lifestyles**		
**Smoking in lifetime**		
No	1.0	
Yes	1.08 (0.48, 2.46)	0.85
**Drinking in the past year**		
No	1.0	
Yes	0.79 (0.35, 1.80)	0.57
**History of HPV screening**		
**HPV screening in the past year**		
No	1.00	
Yes	5.73 (2.35, 13.97)	<0.001
**History of HIV and other sexually transmitted infections (STI) and service utilization**		
**Self-reported HIV sero-status**		
Negative	1.0	
Positive	7.66 (1.04, 56.44)	0.046
Refuse to disclose	1.28 (0.15, 10.98)	0.82
Had never tested for HIV antibody	0.70 (0.09, 5.59)	0.73
**History of other STIs (Yes)**		
No	1.0	
Yes	1.65 (0.76, 3.61)	0.21
**Utilization of other HIV/STI prevention services (e.g., receiving free condoms, peer education and pamphlets, and attending seminars)**		
No	1.0	
Yes	1.85 (0.86, 3.98)	0.12
**Sexual behaviors in the past 6 months**		
**Anal intercourse with regular male sex partners**		
No	1.0	
Yes	0.67 (0.27, 1.67)	0.39
**Anal intercourse with non-regular male sex partners**		
No	1.0	
Yes	1.33 (0.64, 2.76)	0.44
**Anal intercourse with male sex workers**		
No	1.0	
Yes	2.48 (0.25, 24.56)	0.44
**Condomless anal intercourse with men**		
No	1.0	
Yes	0.86 (0.42, 1.78)	0.68
**Multiple male sex partnerships**		
No	1.0	
Yes	1.98 (0.92, 4.27)	0.08
**Sexual intercourse with female sex partners**		
No	1.0	
Yes	3.73 (0.33, 42.36)	0.29
**Condomless sex with female sex partners**		
No	1.0	
Yes	7.50 (0.46, 122.87)	0.16
**Sexualized drug use (use of psychoactive substances before or during sexual intercourse)**		
No	1.0	
Yes	0.97 (0.21, 4.46)	0.97

*CI, Confidence Interval*.

*HIV, Human Immunodeficiency Virus; HPV, Human Papillomavirus; N.A., Not Applicable; OR, Crude Odds Ratio; STI, Sexually Transmitted Infection*.

After adjusting for these two significant sociodemographic characteristics, receiving HPV vaccination during the study period was associated with higher uptake of HPV screening during the same period (AOR: 7.03, 95% CI: 3.07–16.13, *p* < 0.001). In addition, participants who had a higher perceived susceptibility to HPV at baseline (AOR: 1.16, 95% CI: 1.00–1.34, *p* = 0.046) were also more likely to take up HPV screening during the follow-up period (Table 3).

**Table 3:** Associations between perceptions related to HPV screening (in general) and HPV vaccination with the uptake of HPV screening (among participants who completed Month 12 follow-up, *n* = 274).

	**OR (95%CI)**	***P*-values**	**AOR (95%CI)**	***P*-values**
Perceived susceptibility scale	1.21 (1.05, 1.39)	0.009	1.16 (1.00, 1.34)	0.046
Perceived severity scale	1.09 (0.97, 1.23)	0.16	1.08 (0.95, 1.23)	0.23
Perceived benefit scale	0.97 (0.78, 1.21)	0.79	1.00 (0.79, 1.28)	0.99
Perceived barrier scale	0.88 (0.70, 1.12)	0.30	0.90 (0.70, 1.15)	0.39
Cue to action	1.39 (0.97, 1.97)	0.07	1.29 (0.90, 1.85)	0.17
Perceived self-efficacy	0.95 (0.66, 1.36)	0.78	0.90 (0.62, 1.32)	0.61
**Behavioral intention to take up HPV screening**				
Perceived chance to take up clinician-collected HPV screening in the next year if it cost HK $700 (US $89.7) per episode				
Very low/low/moderate	1.0		1.0	
High/very high	1.80 (0.80, 4.03)	0.16	1.67 (0.71, 3.97)	0.24
**Perceived chance to take up clinician-collected HPV screening in the next year if it is provided for free**				
Very low/low/moderate	1.0		1.0	
High/very high	1.66 (0.61, 4.49)	0.32	1.21 (0.43, 3.42)	0.72
**Uptake of HPV vaccination during the study period**				
No	1.00			
Yes	7.77 (3.54, 17.03)	<0.001	7.03 (3.07, 16.13)	<0.001

*AOR, Adjusted Odds Ratio, odds ratios adjusted for significant baseline background characteristics (i.e., self-reported HIV sero-status, and HPV screening in the past year)*.

*CI, Confidence Interval; HPV, Human Papillomavirus; OR, Crude Odds Ratios*.

At Month 12, 46 participants had taken up at least one dose of HPV vaccination. Among the participants who had taken up HPV vaccination or HPV screening during the follow-up period (*n* = 62), 25.8% (*n* = 16) took up HPV screening only, 27.4% (*n* = 17) took up HPV screening together with HPV vaccination, and 46.8% (*n* = 29) received HPV vaccination only.

## Discussion

The interventions promoting HPV vaccination had limited impact in increasing HPV screening among the participants. The uptake of HPV screening during the follow-up period (12.0%) was similar to that reported by the participants 12 months prior to the baseline survey (9.9%). Such uptake rate was much lower than an intervention promoting free HPV screening conducted among MSM in the United States (85%) (35). There are some possible reasons to explain the discrepancy. First, the focus of our interventions was to promote HPV vaccination uptake instead of HPV screening. Second, we did not provide any subsidy for taking up HPV screening, while the study in the United States provided free HPV screening to the study participants (35). Cost was reported as a barrier to HPV screening among MSM (36). Moreover, it was likely that Coronavirus Disease 2019 (COVID-19) had a negative impact on HPV screening uptake as this study was conducted after the outbreak. A study in Hong Kong showed that disruptions in work due to COVID-19 and concerns about the risk of COVID-19 were associated with increased difficulties in accessing HIV and sexual health services (37).

The hypothesis that MSM in Hong Kong would use HPV screening as an alternative prevention strategy to HPV vaccination was not supported by our results. Among those who had taken up HPV screening or HPV vaccination during the study period, about half of them preferred using HPV vaccination alone, and a quarter of them used HPV screening together with HPV vaccination. Very few of them used HPV screening alone. Similar preference was observed among females, as HPV vaccination was considered as a one-off investment with long-term protection (38). Therefore, future programs preventing HPV-related diseases among MSM in Hong Kong should focus on HPV vaccination promotion. Offering free or subsidized HPV vaccines to local MSM is expected to largely increase the uptake rate. Pilot programs providing free HPV vaccination in Australia and the United Kingdom were shown to greatly increase HPV vaccination coverage among MSM (23–25).

It was found that HIV-positive MSM were more likely to take up HPV screening. This echoed the findings of previous studies (35, 39). Given the association between HIV and HPV, HIV-positive MSM are more likely to perceive HPV-related diseases as a serious threat to their health. HPV screening and HPV vaccination should be included as components of the care for HIV-positive MSM. Since the risk of HPV-related diseases among HIV-negative MSM is still much higher than the male general population, attention should be paid to this group to increase their awareness. Having HPV screening in the past year at the baseline was positively associated with HPV screening during the follow-up period. Therefore, it is pivotal to help MSM initiate their first attempt to take up HPV screening in order to become regular screeners. It was also found that the baseline measurement of perceived susceptibility significantly predicted HPV screening uptake during the follow-up period. This demonstrated that MSM with a high perceived susceptibility to HPV infection wanted to be screened to protect themselves from HPV-related diseases. This was in line with the findings of previous studies for HPV vaccination among MSM (30, 39). Furthermore, receiving HPV vaccination was associated with higher uptake of HPV screening. Uptake of HPV vaccination and screening were likely to predict each other. MSM attempted to take both primary and secondary prevention measures to protect themselves.

This study had the strengths of validated data for the uptake of HPV screening, and being one of the first interventional studies related to HPV screening among Chinese MSM. It also had several limitations. First, there was a selection bias since we were unable to collect information from MSM who refused to participate. They might have different characteristics compared to the participants. Second, since the project aimed to evaluate this service model in real-world settings, we did not have a control or comparison group. As compared to randomized controlled trials, such evaluation design has little control over issues affecting internal validity. To our knowledge, the availability and cost of HPV screening for MSM in Hong Kong did not change in the past decade. There were no programs promoting HPV screening or vaccination for MSM in Hong Kong before this service model was implemented, and none of the study participants were exposed to other HPV vaccination/screening promotions during the project period. Therefore, we believed the difference in HPV screening uptake between this study and historical record could be mainly attributed to our service model. Third, attrition bias might exist. Participants who dropped out at Month 12 were more likely to have high-risk behaviors at baseline (e.g., anal intercourse with non-regular male sex partners and male sex workers). MSM with high-risk behaviors might be beneficial more from the intervention. This might partially explain the ineffectiveness of the intervention in increasing HPV screening since lots of high-risk individuals were dropped out. Fourth, since this study was based on a convenient sample of MSM, generalizing the results to MSM in Hong Kong should be made with caution.

## Conclusions

The CBO-private clinic service model promoting HPV vaccination had limited impact in increasing HPV screening among MSM in Hong Kong. More participants preferred HPV vaccination or HPV vaccination plus HPV screening, and very few of them preferred HPV screening alone. The hypothesis that MSM in Hong Kong would use HPV screening as an alternative prevention strategy to HPV vaccination was not supported by the data. Future programs preventing HPV-related diseases among MSM in Hong Kong should focus on HPV vaccination promotion.

## Data Availability Statement

The data presented in this study are available from the corresponding author upon request. The data are not publicly available as they contain sensitive personal behaviors.

## Ethics Statement

The studies involving human participants were reviewed and approved by the Survey and Behavioral Research Ethics Committee of the Chinese University of Hong Kong (reference number: KPF18HLF22 and date of approval: 18 March 2019). Informed consent was obtained from all participants involved in the study.

## Author Contributions

ZW, YF, AC, and FF: conceptualization. PC, ZW, and MI: methodology: MI: data curation and project administration. PC and ZW: formal analysis and writing-review and editing. PC, YF, and ZW: writing-original draft preparation. All authors have read and agreed to the published version of the manuscript.

## Funding

This research was funded by the Knowledge Transfer Project Fund, the Chinese University of Hong Kong (grant number: KPF18HLF22).

## Conflict of Interest

The authors declare that the research was conducted in the absence of any commercial or financial relationships that could be construed as a potential conflict of interest.

## Publisher's Note

All claims expressed in this article are solely those of the authors and do not necessarily represent those of their affiliated organizations, or those of the publisher, the editors and the reviewers. Any product that may be evaluated in this article, or claim that may be made by its manufacturer, is not guaranteed or endorsed by the publisher.
